# Multi-localization transport behaviour in bulk thermoelectric materials

**DOI:** 10.1038/ncomms7197

**Published:** 2015-02-04

**Authors:** Wenyu Zhao, Ping Wei, Qingjie Zhang, Hua Peng, Wanting Zhu, Dingguo Tang, Jian Yu, Hongyu Zhou, Zhiyuan Liu, Xin Mu, Danqi He, Jichao Li, Chunlei Wang, Xinfeng Tang, Jihui Yang

**Affiliations:** 1 State Key Laboratory of Advanced Technology for Materials Synthesis and Processing, Wuhan University of Technology, Wuhan 430070, China; 2 Materials Science and Engineering Department, University of Washington, Seattle, Washington 98195, USA; 3 School of Physics, State key Laboratory of Crystal Materials, Shandong University, Jinan 250100, China

## Abstract

Simultaneously optimizing electrical and thermal transport properties of bulk thermoelectric materials remains a key challenge due to the conflicting combination of material traits. Here, we have explored the electrical and thermal transport features of In-filled CoSb_3_ through X-ray absorption fine structure, X-ray photoemission spectra, transport measurement and theoretical calculation. The results provide evidence of three types of coexisting multi-localization transport behaviours in the material; these are heat-carrying phonon-localized resonant scattering, accelerated electron movement and increase in density of states near the Fermi level. The 5*p*-orbital hybridization between In and Sb is discovered in the In-filled CoSb_3_ compound, which results in a charge transfer from Sb to In and the enhancement of *p–d* orbital hybridization between Co and Sb. Our work demonstrates that the electrical and thermal properties of filled skutterudite bulk thermoelectric materials can be simultaneously optimized through the three types of coexisting multi-localization transport behaviours in an independent way.

## Abstract (web summary)

An ideal thermoelectric material has a large Seebeck coefficient, and a high electrical but low thermal conductivity; however, optimizing all three is difficult. Zhao *et al*. discover three types of coexisting multi-localization transport behaviours in filled skutterudite materials that aid this optimization.

## Graphical abstract

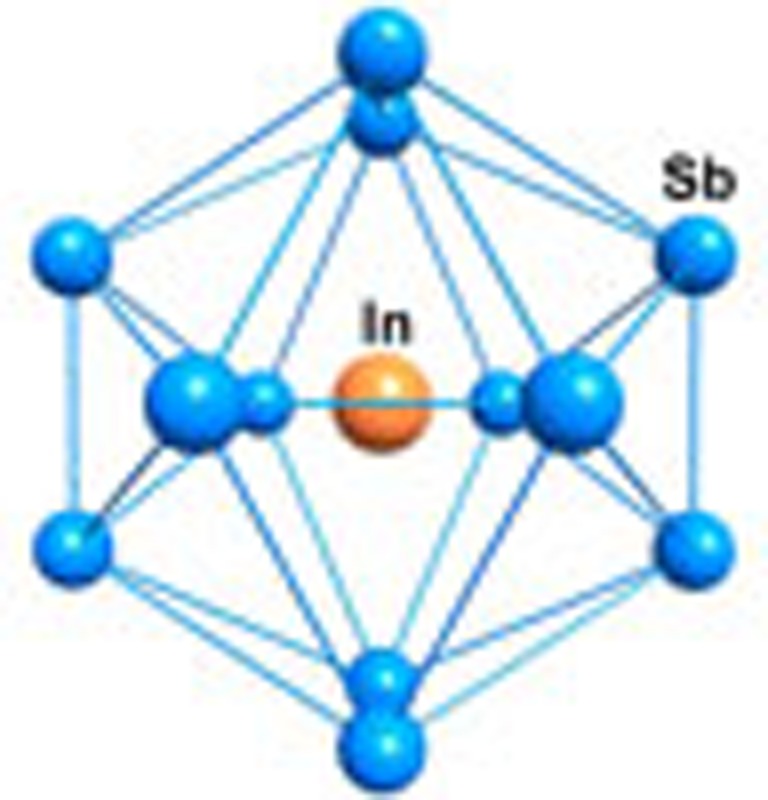

Thermoelectric (TE) devices, which can directly convert heat into electricity and vice versa, have attracted considerable attention due to a variety of applications in heating, cooling, power generation and waste heat recovery^1^. Their conversion efficiency depends on the dimensionless figure of merit of TE materials defined as *ZT=α*^2^*σT/κ*, where *T* is the absolute temperature, *σ* is the electrical conductivity, *α* is the Seebeck coefficient and *κ* is the total thermal conductivity (*κ=κ*_E_*+κ*_L_, where *κ*_E_ is the electronic contribution and *κ*_L_ the lattice contribution). Numerous efforts have been attempted to improve *ZT* in the past two decades despite a compromise of *κ* and *α* with *σ* in TE materials^2^. To decrease *κ*_L_, various approaches used to enhance phonon scattering have taken advantage of nanoinclusion^3^,^4^,^5^,^6^, alloying^7^, rattling filler^8^, quasi-ballistic transport nanoscale interfaces or nanopores^9^,^10^, liquid-like behaviour copper ions^11^ and anharmonic phonon coupling^12^. Meanwhile, a series of band structure engineering approaches such as high valley degeneracy^13^,^14^, peierls distortion^15^, electron energy filtering near the Fermi level^16^,^17^,^18^ and optimal bandwidth^19^, have been employed to improve the electrical properties. Some important single-localization transport behaviours have been discovered in different TE materials. For example, interface scattering in AgPb_*m*_SbTe_2__+*m*_ (ref. 4) and BiSbTe (ref. 6), and localized resonant scattering in filled CoSb_3_ (ref. 8) have remarkably enhanced phonon scattering and reduced *κ*_*L*_; band convergence in PbTe_1−*x*_Se_*x*_ (ref. 13) and Mg_2_Si_1−*x*_Sn_*x*_ (ref. 14), charge density wave in In_4_Se_3_ (ref. 15) and electron resonant state in PbTe (ref. 16) have all led to an effective increase in *α*. These single-localization transport behaviour, however, can only optimize a single physical parameter of electrical or thermal properties. So far, it remains a major challenge to simultaneously increase *α* and *σ* while reducing *κ*, because no material has been found that shows multiple-localization transport behaviour.

Filled CoSb_3_ have been intensely pursued as an important TE material for intermediate-temperature power generation. The major progress in improving *ZT* has made through decreasing *κ*_L_ by filling the icosahedron voids in CoSb_3_ with foreign atoms (for example, rare earths, alkali earths or alkali metals) to enhance heat-carrying phonon-localized resonant scattering via filler rattling^8^,^20^,^21^,^22^,^23^,^24^,^25^,^26^,^27^,^28^,^29^. Shi *et al*.^28^ suggested that the electrical properties of multiple-filled CoSb_3_ could be optimized by adjusting the total filling fraction of fillers with different charge states. However, the tuning space of electrical properties is limited due to the conflicting relationship among *α*, n and *σ*, as expressed in the following formulae:


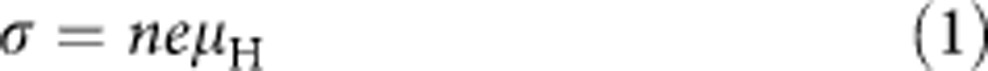



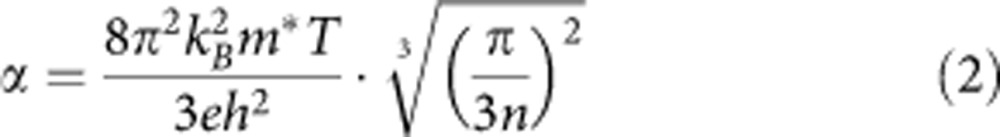


where m* is the carrier effective mass; *n*, the carrier concentration and *μ*_H_, the carrier mobility. Recently, more and more experiments indicate that group III elements (Ga, In and Tl) can remarkably improve *ZT* of CoSb_3_ materials because of an almost perfect combination of low *κ*_L_, high *σ* and large *α*^30^,^31^,^32^,^33^,^34^,^35^,^36^,^38^. However, it still remains unsettled how the group III elements synergistically adjust the electrical and thermal properties of CoSb_3_. The Tl filler rattling only explains the low *κ*_L_ of Tl-filled CoSb_3_ (refs 30, 31). The dual-site occupancy at both the voids and Sb sites for Ga in CoSb_3_ is only responsible for low *κ*_L_ and *n*^32^. Up to now, the doping behaviour of the In impurity in CoSb_3_ remains an ongoing debate^33^,^34^,^35^,^36^,^38^,^39^,^40^,^41^,^42^.

In the following, we have explored the electrical and thermal transport features of In-filled CoSb_3_ through X-ray absorption fine structure (XAFS), X-ray photoemission spectra (XPS), transport measurement and theoretical calculation. Our data suggest that there are three types of coexisting multi-localization transport behaviours including heat-carrying phonon-localized resonant scattering, accelerated electron movement and increase in density of states (DOSs) near the Fermi level. Our work demonstrates that the electrical and thermal properties can be independently optimized through the three types of coexisting multi-localization transport behaviours.

## Results

### Filling behaviour of In impurity in CoSb_3_

**Figure 1:**
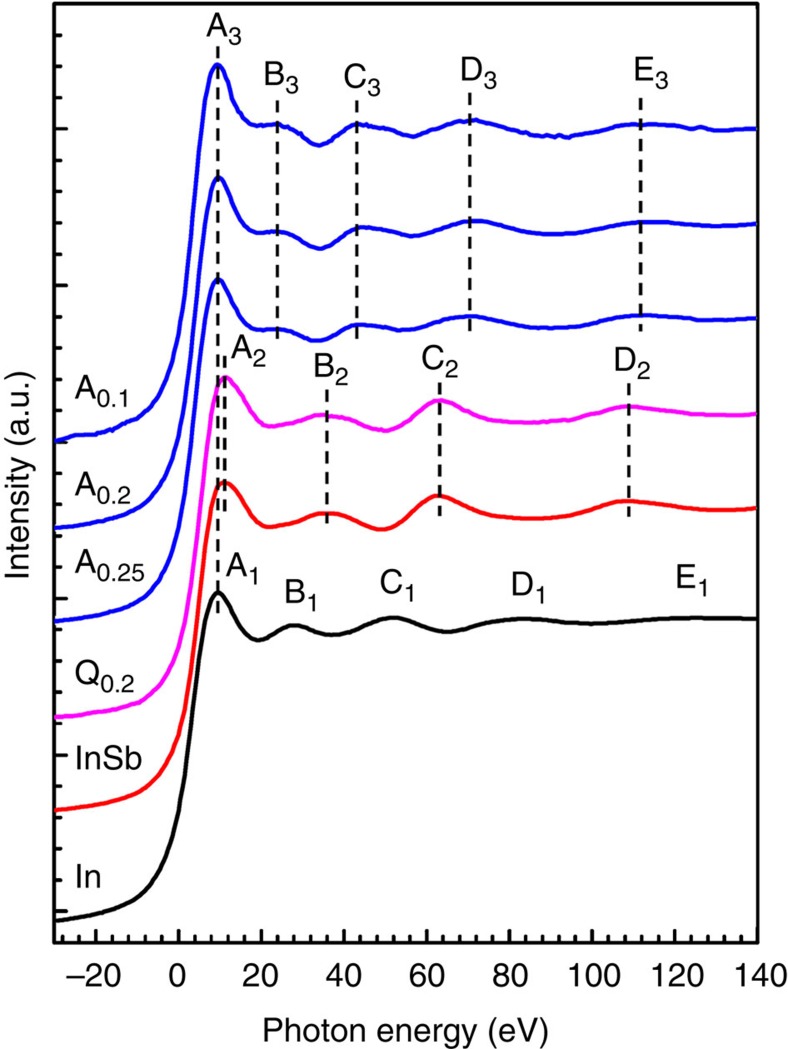
The In *K*-edge XANES experimental spectra. The quenched In_0.2_Co_4_Sb_12_ is symbolized with ‘Q_0.2_’. The annealed In_*x*_Co_4_Sb_12_ (*x*=0.1, 0.2 and 0.25) is symbolized with ‘A_*x*_’. The In *K*-edge XANES experimental spectra of InSb and In metals are plotted for comparison. Zero eV corresponds to the threshold of In *K*-edge (27,940 eV).

We compare In *K*-edge X-ray absorption near-edge structure (XANES) experimental spectra of quenched In_0.2_Co_4_Sb_12_ (Q_0.2_) and annealed In_*x*_Co_4_Sb_12_ (*x*=0.1, 0.2 and 0.25) (A_*x*_) with those of InSb and In metal (Fig. 1). The In *K*-edge XANES spectrum of the In metal has five absorption peaks A_1_, B_1_, C_1_, D_1_ and E_1_ centred at about 9, 28, 52, 84 and 128 eV, respectively, whereas that of InSb has four absorption peaks A_2_, B_2_, C_2_ and D_2_ at about 11, 35, 64 and 107 eV, respectively. The In *K*-edge XANES spectrum of Q_0.2_ has four absorption peaks with almost the same positions as those of InSb, indicating the existence of InSb in Q_0.2_.

All the In *K*-edge XANES spectra of the A_*x*_ samples encompass five absorption peaks A_3_, B_3_, C_3_, D_3_ and E_3_ with energy near 9, 23, 42, 70 and 113 eV, respectively. The main peak A_3_ has the same energy as that of A_1_ for the In metal, but is 2 eV lower than that of A_2_ for InSb. For the In *K*-edge XANES spectra, the main peaks A_1_, A_2_ and A_3_ can be attributed to the 1*s*→5*p* transition. The energy discrepancy of A_2_ and A_3_ indicates that the chemical states of the In impurity are different between the A_*x*_ samples and InSb. It is worth noting that the absorption peaks B_3_, C_3_, D_3_ and E_3_ of all the A_*x*_ samples are distinctly different in energy from B_1_, C_1_, D_1_ and E_1_ for the In metal, and from B_2_, C_2_ and D_2_ of InSb. Such significant differences undoubtedly show that the In impurities in the A_*x*_ samples are neither InSb nor the In metal. Accordingly, it is highly plausible that the In impurities have been incoporated in the lattice of CoSb_3_ in all annealed samples, well consistent with the X-ray diffraction results (see Supplementary Fig. 1).

**Figure 2:**
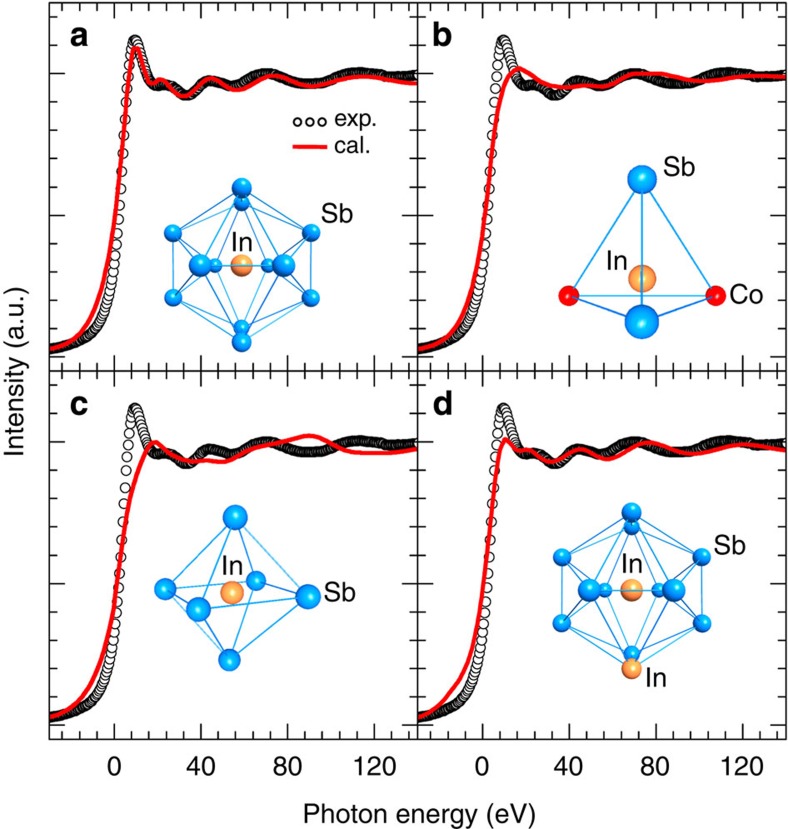
The In *K*-edge XANES spectra of In impurity in CoSb_3_. (**a**) Filling Sb_12_ icosahedron voids at the 2*a* sites, (**b**) substituting for Sb at the 24*g* sites, (**c**) substituting for Co at the 8*c* sites and (**d**) simultaneously filling the icosahedron voids at the 2*a* sites and substituting for Sb at the 24*g* sites. The In *K*-edge XANES experimental spectra of A_0.25_ are plotted for comparison. The XANES theoretical spectra are shown with red solid lines and symbolized as ‘cal.’. The XANES experimental ones are shown with black circle lines and symbolized as ‘exp.’.

Because of the close electronegativity values among In (1.78), Sb (2.05) and Co (1.88), there exist four possible occupational sites for the In impurities in CoSb_3_, filling the icosahedron voids at the 2*a* sites to form In-filled CoSb_3_, substituting for Sb at the 24*g* sites in the disordered Sb_2_Co_2_ tetrahedron to form In-doped CoSb_3−*s*_In_*s*_, substituting for Co at the 8*c* sites in the irregular Sb_6_ octahedron to form In-doped Co_1−*r*_In_*r*_Sb_3_ or simultaneously filling the icosahedron voids at the 2*a* sites and substituting for Sb at the 24*g* sites to form (In_VF_)_*x*2/3_Co_4_Sb_12−*x*/3_(In_Sb_)_*x*/3_ with charge-compensated compound defects^33^. The In *K*-edge XANES theoretical spectra of the In impurities at the 2*a*, 24*g*, 8*c* and 2*a*–24*g* sites in CoSb_3_ were calculated to identify which sites the In impurities occupy. The In *K*-edge XANES theoretical spectra (red solid lines symbolize ‘cal.’) of In-filled CoSb_3_ for the In impurities (a) filling icosahedron voids at the 2*a* sites, (b) substituting for Sb at the 24*g* sites, (c) substituting for Co at 8*c* sites and (d) simultaneously filling the icosahedron voids at the 2*a* sites and substituting for Sb at the 24*g* sites are compared with the experimental spectrum (circle lines symbolize ‘exp.’) of the A_0.25_ sample (Fig. 2). It is clear that only the In *K*-edge XANES theoretical spectrum for filling icosahedron voids is in good agreement with the experimental data; the other three cases have large discrepencies between the theoretical spectra and the experimental ones (Fig. 2b–d). Therefore, the In *K*-edge XANES spectra unequivocally suggest that the In impurities stably fill the Sb_12_ icosahedron voids in CoSb_3_.

### 5*p*-orbital hybridization between In and Sb and its effects

**Figure 3:**
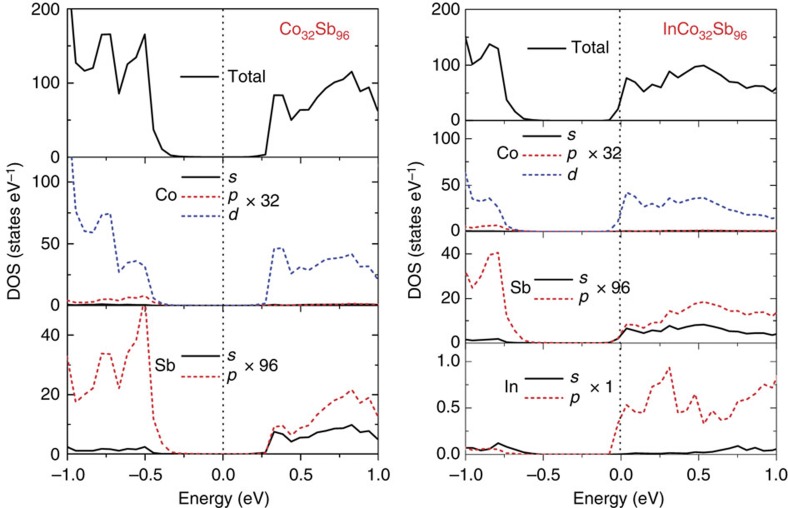
Total DOS and partial DOS near VBM and CBM of CoSb_3_ and In_0.125_Co_4_Sb_12_. The 2 × 2 × 2 supercells were calculated using projector-augmented wave method implemented in CASTEP package based on the density functional theory.

**Figure 4:**
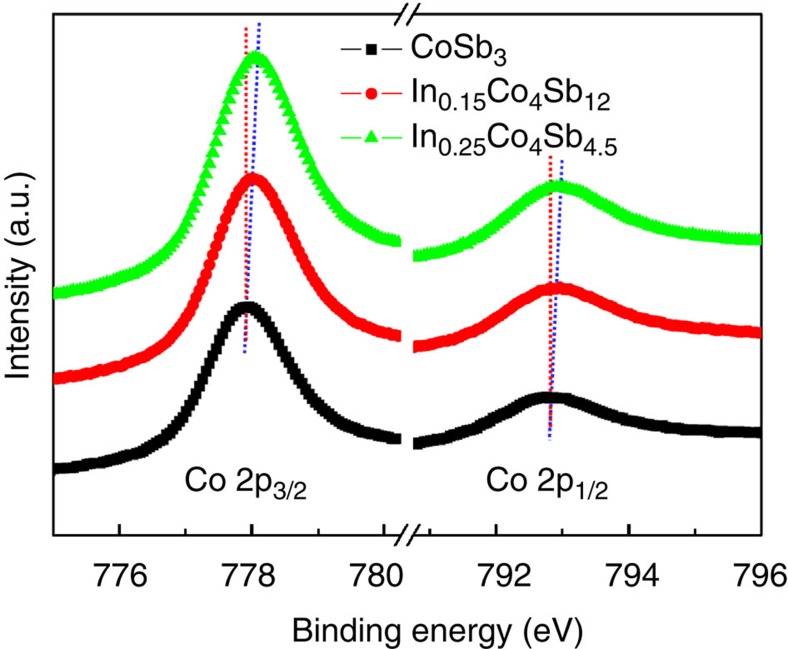
XPS spectra of Co 2*p*_3/2_ and 2*p*_1/2_ core levels for CoSb_3_ and In-filled CoSb_3_. Measurements were performed under the CAE mode with pass energy of 25 eV, step size of 0.05 eV and 128 scans with a Thermo VG Multilab 2000 spectrometer.

The total DOSs of CoSb_3_ and In_0.125_Co_4_Sb_12_, and partial DOS for Co, Sb and In atoms indicate that the total DOS of In-filled In_0.125_Co_4_Sb_12_ near valence band maximum (VBM) and conduction band minimum (CBM) mainly stem from Co 3*d* electrons and Sb 5*p* electrons (Fig. 3). It can be seen that there is an extra peak of the partial DOS of Co 3*d* and Sb 5*p* electrons near 0.31 eV for In_0.125_Co_4_Sb_12_, which exactly corresponds to the highest peak of the partial DOS of In 5*p* electrons. Particularly, the DOS_Sb5*p*_/DOS_Co3*d*_ ratio is decreased near VBM from 2.96 for CoSb_3_ to 2.08 for In_0.125_Co_4_Sb_12_ and increased near CBM from 0.20 for CoSb_3_ to 0.22 for In_0.125_Co_4_Sb_12_. This evolution suggests that the energy of Sb 5*p* electrons and Co 3*d* electrons becomes closer, and the *p–d* orbital hybridization between Co and Sb has thus been enhanced in In_0.125_Co_4_Sb_12_. Experimentally, the XPS spectra of Co 2*p*_3/2_ and 2*p*_1/2_ core levels of In-filled CoSb_3_ are gradually shifted to higher binding energies (maximum up to 0.20 eV) as the filling fraction of the In filler increased (Fig. 4). The chemical shift is less than the energy resolution of XPS (about 0.47 eV) due to too low filling fraction of the In filler; however, the chemical shift can be repeated (see Supplementary Fig. 2) and thus may provide a plausible evidence of enhanced *p–d* orbital hybridization between Co and Sb.

**Figure 5:**
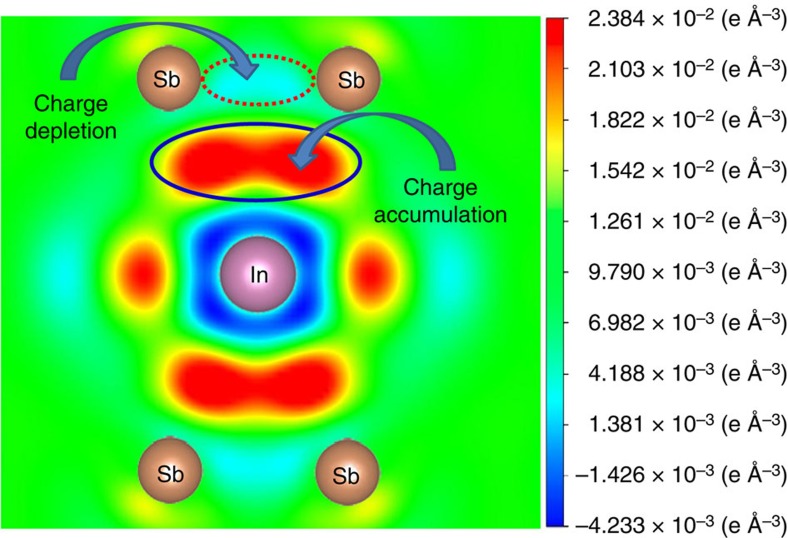
Differential charge density of In_0.125_Co_4_Sb_12_ projected on the (111) plane. The 2 × 2 × 2 supercells were calculated using the projector-augmented wave method implemented in CASTEP package based on the density functional theory. The differential charge density. delta *p*=*p*_InCo32Sb96_−*p*_Co32Sb96_−*p*_In_

To clarify the origin of enhanced *p–d* orbital hybridization between Co and Sb in In-filled CoSb_3_, the partial DOS of In atoms in the range of −12~2 eV have been analysed. We discover that the partial DOS of In 5*s* electrons are distributed about 1.0 eV below the Fermi level (see Supplementary Fig. 3). Therefore, all In 5*s* electrons are confined at the deep locations of the valence band and have no contribution to *n*. Although there are a few In 5*p* electrons below the Fermi level, the partial DOS of In 5*p* electrons are mainly distributed above and near the Fermi level, suggesting that the In 5*p* electrons are almost lost in In-filled CoSb_3_. The electronic states of the In impurity clearly show that the effective charge of the In filler is smaller than, but very close to, +1. Therefore, the electronic configuration of the In filler is 5*s*^2^4*d*^10^5*p*^0^, suggesting that the In filler may provide three unoccupied 5*p* orbitals for a 5*p*-orbital hybridization between In and Sb. This is corroborated by the differential charge density of In_0.125_Co_4_Sb_12_ projected on the (111) plane (Fig. 5) clearly showing the 5*p*-orbital hybridization between In and Sb in In-filled CoSb_3_. Therefore, the enhancement in *p–d* orbital hybridization between Co and Sb in In-filled CoSb_3_ must originate from the 5*p*-orbital hybridization between In and Sb. Note that the charge density decreases between Sb and Sb atoms while it increases between In and Sb atoms for In-filled icosahedron voids, indicating that the partial charges are transferred from Sb to In, which are in good agreement with our previous XPS results^27^. Namely, the 5*p*-orbital hybridization between In and Sb in In-filled CoSb_3_ can still cause a charge transfer from Sb to In and produce two types of atomic-scale electric fields near the In-filled Sb_12_ icosahedron, which are the atomic-scale electric fields with positive charge at the framework of In-filled Sb_12_ icosahedron and the atomic-scale electric fields with negative charge in the Sb_12_ icosahedron. Since the framework of Sb_12_ icosahedron acts as the passage of majority carriers (electrons) in In-filled CoSb_3_, the atomic-scale electric fields with positive charge at the framework of In-filled Sb_12_ icosahedron may accelerate electron movement.

### In–Sb weak covalent bond and its effects

The extended XAFS (EXAFS) analysis reveals that the In–Sb bond length is about 3.35 Å for the A_0.2_ sample (see Supplementary Fig. 4) and very close to the value (3.36 Å) reported for In_0.2_Co_4_Sb_12_ (ref. 34), while it is only about 2.81 Å for InSb^43^. The longer In–Sb bond indicates less orbital overlapping and weakened repulsion interaction between bonding and antibonding states of In–Sb bond in In-filled CoSb_3_. Therefore, the In–Sb bond between In filler and host framework of Sb_12_ icosahedron must be a weak covalent bond in In-filled CoSb_3_, further corroborating the lower energy of the main peak A_3_ than that of A_2_ (Fig. 1). Obviously, the In fillers can rattle inside the voids and cause heat-carrying phonon-localized resonant scattering, thereby remarkably reducing *κ*_L_.

**Figure 6:**
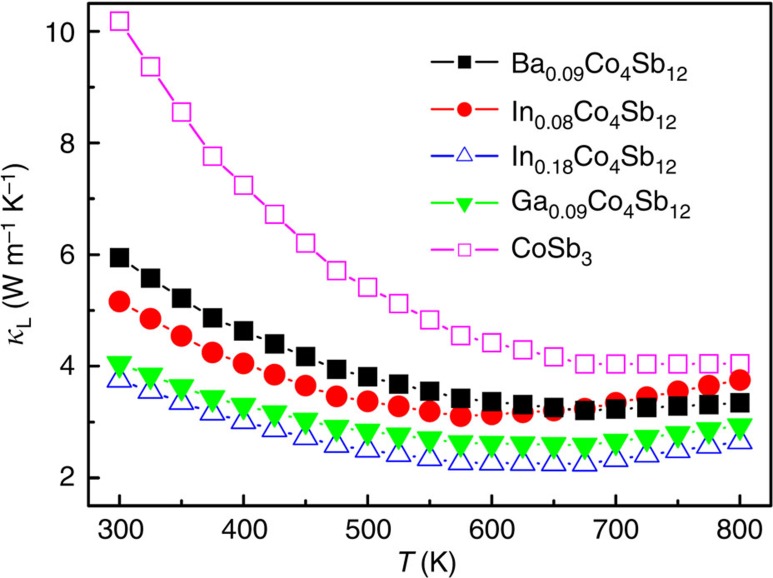
Temperature dependences of lattice thermal conductivity in the range of 300–800 K. The data of CoSb_3_ are plotted for comparison with those of In-, Ba-, and Ga-filled CoSb_3_.

The temperature dependences of *κ*_L_ values for CoSb_3_ and In-, Ba- and Ga-filled CoSb_3_ (Fig. 6) show that *κ*_L_ value at 300 K is only about 5.16 W m^−1^ K^−1^ for In_0.08_Co_4_Sb_12_ and 3.75 W m^−1^ K^−1^ for In_0.18_Co_4_Sb_12_ while more than 10 W m^−1^ K^−1^ for CoSb_3_. The *κ*_L_ values of In_0.08_Co_4_Sb_12_ are smaller than those of Ba_0.09_Co_4_Sb_12_ in the range of 300–650 K, suggesting that the In filler is more effective in reducing *κ*_L_ than Ba at a comparable filling fraction. The *κ*_L_ values of In_0.08_Co_4_Sb_12_, however, are significantly greater than those of Ga_0.09_Co_4_Sb_12_ in the range of 300–800 K, suggesting different doping behaviour in CoSb_3_ between In and Ga. The lower *κ*_L_ values of Ga_0.09_Co_4_Sb_12_ are due to the additional defect scattering induced by the Sb-substitutional Ga, because Ga impurties in CoSb_3_ were thought to simultaneously occupy both the icosahedron voids and the Sb sites^32^.

## Discussion

**Figure 7:**
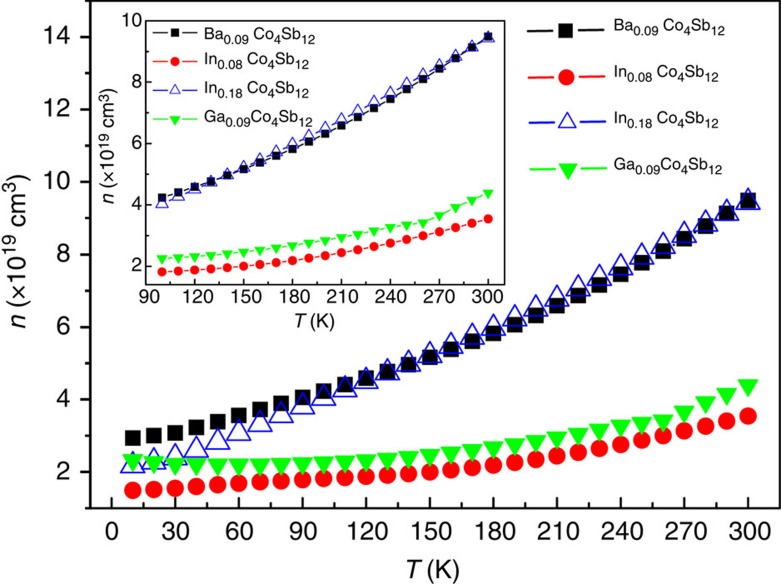
Temperature dependences of carrier concentration in the range of 10–300 K. The inset shows the temperature dependences of carrier concentration of In-, Ba-, and Ga-filled CoSb_3_ in the range of 100–300K.

**Figure 8:**
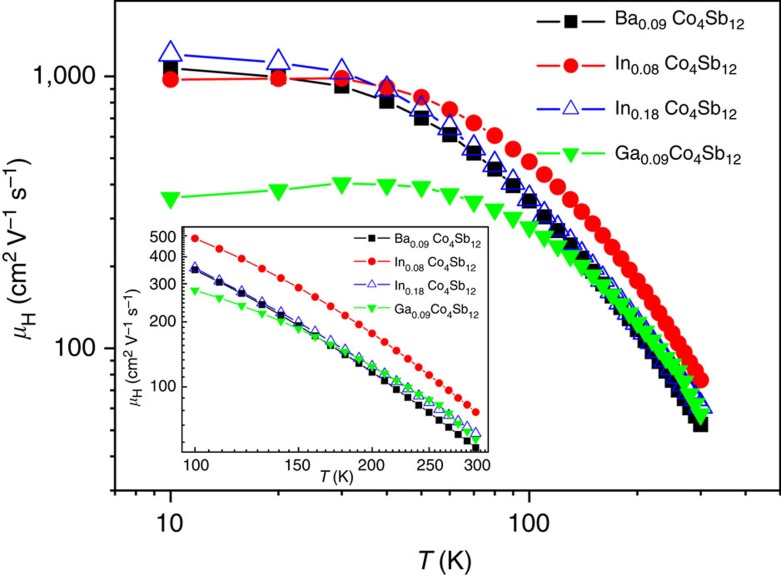
Temperature dependences of Hall mobility in the range of 10–300 K. The inset shows the temperature dependences of Hall mobility of In-, Ba-, and Ga-filled CoSb_3_ in the range of 100–300K.

**Figure 9:**
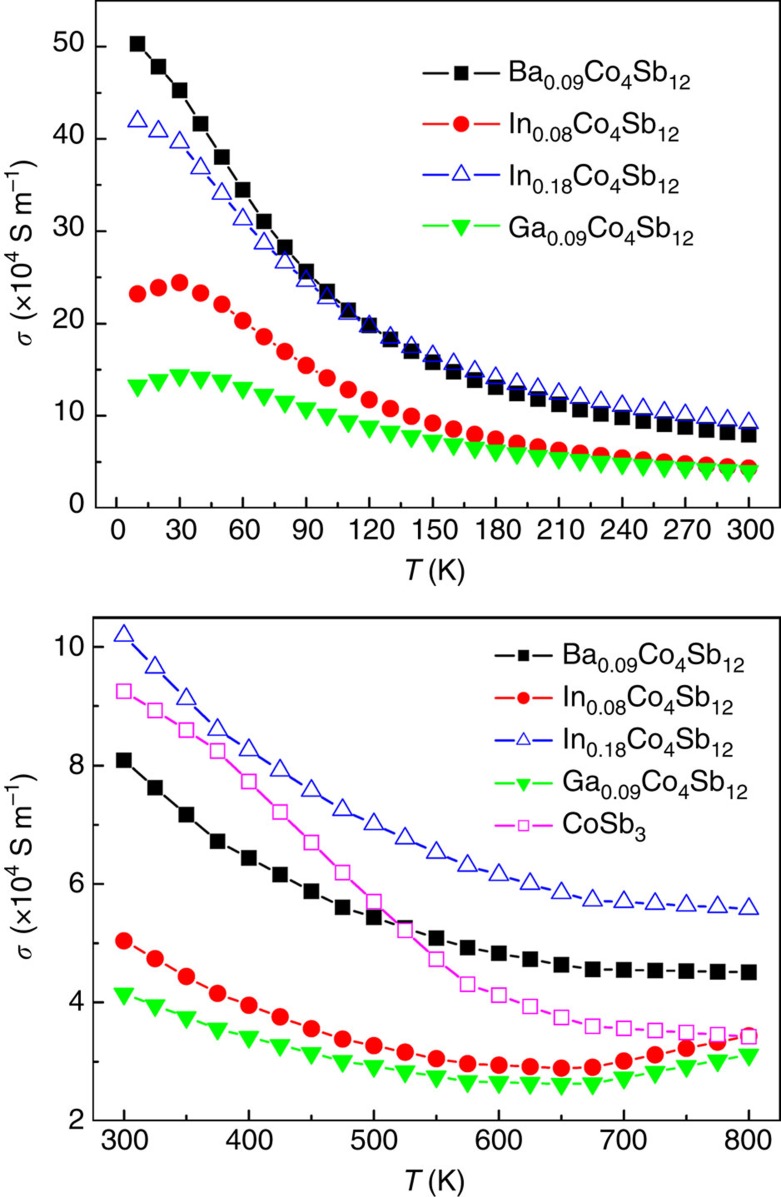
Temperature dependences of electrical conductivity in the range of 10–800 K. The data of CoSb_3_ in the range of 300–800K are plotted for comparison with those of In-, Ba-, and Ga-filled CoSb_3_.

The *n* values of In_0.18_Co_4_Sb_12_ are almost the same as those of Ba_0.09_Co_4_Sb_12_ in the range of 100–300 K (Fig. 7), clearly indicating that the In filler provides one electron and is univalent (In^+^) in In-filled CoSb_3_ because the Ba filler provides two electrons in Ba-filled CoSb_3_. The electronic structure of the In filler described above not only is the physical mechanism of low *n* for In-filled CoSb_3_, but also may reasonably explain why the *n* values of In_0.18_Co_4_Sb_12_ and Ba_0.09_Co_4_Sb_12_ are very close in the range of 100–300 K. At the same time, the charge transfer from Sb to In in In-filled CoSb_3_ must produce the same amount of atomic-scale electric fields with positive charge at the framework of In-filled Sb_12_ icosahedron; therefore, the major carriers (electrons) nearby the In-filled Sb_12_ icosahedron are not only partially annihilated but also accelerated because of the attraction by the atomic-scale electric fields with positive charge. These multi-functional local transport effects may explain that the In-filled CoSb_3_ has higher *μ*_H_ than those of Ba-filled and Ga-filled CoSb_3_ in the range of 100–300 K under comparable *n* values (Fig. 8). As a result, the In-filled CoSb_3_ have higher *σ* than Ba- and Ga-filled CoSb_3_ in the range of 150–800 K, although their *n* values are very close (Fig. 9), which can be attributed to an increase in *μ*_H_ induced by accelerated electron movement nearby the In-filled Sb_12_ icosahedron. Compared with Ba- and In-filled CoSb_3_, the lower *μ*_H_ of Ga-filled CoSb_3_ in the range of 10–100 K may be reasonably explained by the dual-site occupancy of Ga impurity in CoSb_3_ (ref. 32). In addition, the *μ*_H_ values of Ba- and In-filled CoSb_3_ share similar temperature dependence in the range of 10–300 K (Fig. 8), implying that the electron scattering mechanisms are the same for both cases and there is thus no case of In occupying at the Sb sites. This is well consistent with the XANES results as shown in Fig. 2.

**Figure 10:**
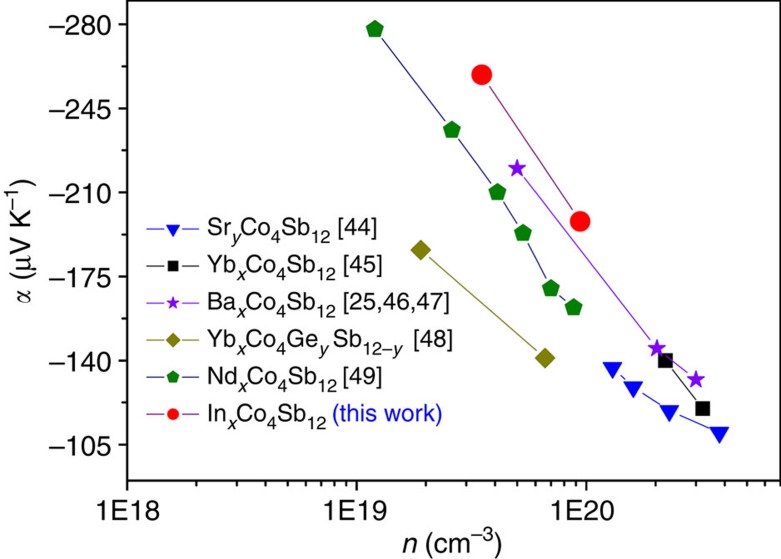
Carrier concentration dependences of the Seebeck coefficient of *n*-type filled CoSb_3_ at room temperature. Here, compare the data of Sr-, Yb-, Ba-, Nd-, and In-filled CoSb_3_ with n on the order of 10^19^~10^20^ cm^−3^.

The enhancement of the *p–d* orbital hybridization between Co and Sb induced by the In filler still provides a more reasonable explanation for the band structure of In_0.125_Co_4_Sb_12_. Compared with CoSb_3_ (see Supplementary Fig. 5), the Fermi level of In_0.125_Co_4_Sb_12_ is migrated into conduction bands and the energy gap between the Fermi level and CBM at *H*, *N* and *P* points with high symmetry is significantly decreased from 0.45~0.35 eV for CoSb_3_ to 0.12~0.03 eV for In_0.125_Co_4_Sb_12_. As a result, the DOS of VBM is significantly decreased while the DOS of CBM is remarkably increased. Namely, there is an asymmetric distribution of DOS near the Fermi level of In-filled CoSb_3_ beneficial to obtaining a large *α*. Such a DOS asymmetric distribution may very well explain why the absolute *α* values of In-filled CoSb_3_ are higher than those of *n*-type Ba-, Sr-, Yb- and Nd-filled CoSb_3_, with similar *n* on the order of 10^19^ cm^−3^ at room temperature^25^,^44^,^45^,^46^,^48^,^49^, as shown in Fig. 10. The *α* values at 300 K reached 259 μV K^−1^ for In_0.08_Co_4_Sb_12_ with 3.5 × 10^19^ cm^−3^ and 198 μV K^−1^ for In_0.18_Co_4_Sb_12_ with 9.4 × 10^19^ cm^−3^. Obviously, the large *α* values of In-filled CoSb_3_ originate from the increase in DOS of CBM near the Fermi level due to enhanced *p–d* orbital hybridization between Co and Sb induced by the In filler.

Therefore, the perfect combination of low *κ*_L_, high *σ* and large *α* for In-filled CoSb_3_ originates from the following physical and chemical mechanisms. First, the low *κ*_L_ is due to the heat-carrying phonon-localized resonant scattering induced by In-filler rattling. Second, the high *σ* is attributed to the accelerated electron movement induced by the charge transfer from Sb to In. Third, the large *α* benefits from the increase in DOS of CBM near the Fermi level induced by the enhanced *p–d* orbital hybridization between Co and Sb. The 5*p*-orbital hybridization between In and Sb in In-filled CoSb_3_ can cause a charge transfer from Sb to In and the enhancement of *p–d* orbital hybridization between Co and Sb. The fundamental origin of low *n* of In-filled CoSb_3_ is that all 5*s* electrons of the In filler are confined at the deep locations of the valence band. The low *n* and asymmetric distribution of DOS near the Fermi level provide a favourable condition for adjusting *σ* and *α* of In-filled CoSb_3_ in an independent way.

## Methods

### Synthesis and characterization

In-filled In_*x*_Co_4_Sb_12_ (*x*=0.1, 0.2 and 0.25) bulk materials were prepared by a combination of melting, annealing and spark plasma sintering reported elsewhere^25^. Another three bulk materials (CoSb_3_, Ba_0.1_Co_4_Sb_12_ and Ga_0.1_Co_4_Sb_12_) were prepared with the same method for comparsion. X-ray diffraction (PANalytical X’ Pert PRO) and scanning electron microscope analysis confirmed that all the annealed samples In_*x*_Co_4_Sb_12_ (*x*=0.1, 0.2 and 0.25) were composed of single-phase skutterudite, while all the quenched samples consisted of Sb, CoSb, CoSb_2_ and InSb. Chemical compositions of all the bulk materials were determined by electron probe microanalysis (EPMA, JXA-8230). XANES and EXAFS of quenched In_0.2_Co_4_Sb_12_ and annealed In_*x*_Co_4_Sb_12_ samples were measured under a working voltage of 3.5 GeV and a working current of 300 mA at BL14W1 beamline in the Shanghai Synchrotron Radiation Facility (SSRF). A Si (311) double-crystal monochromater with energy resolution of 0.5 × 10^−4^ eV@10 keV was employed to measure In *K*-edge spectra. All XANES and EXAFS spectra were measured three times to ensure reproducibility. The In *K*-edge XANES spectra of InSb and the In metal were also recorded for comparison. XPS of Co 2*p*_3/2_ and 2*p*_1/2_ core levels were recorded at pass energy of of 25 eV, step size of 0.05 eV and 128 scans with Thermo VG Multilab 2000 spectrometer.

### Transport measurement

The *σ* and *α* values were measured with the standard four-probe method (UlvacRiko: ZEM-3) in Ar atmosphere. The *κ* was calculated using the equation *κ=DρC*_p_, where *C*_p_ is the specific heat capacity, *ρ* the bulk density and *D* the thermal diffusion coefficient. *D* was measured by a laser flash technique (Netzsch LFA 427) in a flowing Ar atmosphere, *C*_p_ with a TA Q20 differential scanning calorimeter and *ρ* by Archimedes method. *κ*_L_ was obtained by subtracting the electrical contribution from *κ* using the equation *κ*_L_=*κ*−*κ*_E_. *κ*_E_ is expressed by the Wiedemann–Franz *κ*_E_=*σLT*, where *L* is the Lorenz number. Uncertainties are ±5–7% for *σ* and *κ*_L_, and ±5% for *α*. The *n* and *μ*_H_ were measured under 10–300 K with Quantum Design PPMS.

### Theoretical calculation

The *K*-edge XANES theoretical spectra of In impurity at four kinds of crystallographic sites (2*a*, 24*g*, 8*c* and 2*a*–24*g*) in CoSb_3_ were calculated with the self-consistent multiple-scattering theory based on real-space clusters implemented in FEFF9 package^50^. The In *K*-edge EXAFS experimental spectra were first normalized and background subtracted to obtain the *k*-weighted spectra, and then Fourier transformed to obtain the length of the In–Sb bond. The DOSs, band structure and differential charge densities projected on the (111) plane of CoSb_3_ and In_0.125_Co_4_Sb_12_ using a 2 × 2 × 2 supercell were calculated using a projector-augmented wave method implemented in CASTEP package based on the density functional theory^51^. Lattice relaxation and structural optimization were carried out through total energy calculations.

## Author contributions

W.Z., P.W. and Q.Z. designed and carried out XANES and EXAFS experiments. P.W., W.Z., D.T., J.Y., H.Z. and Z.L. synthesized the samples and carried out the thermoelectric properties measurements. H.P., J.L. and C.W. performed the electron structure calculations. W.Z., P.W., W.Z., X.T. and J.Y. performed the Hall measurements. W.Z. and D.T. performed the XPS measurements. W.Z., P.W., Q.Z and J.Y. conceived the experiments, analysed the results and wrote and edited the manuscript. All authors read the paper and commented on the text.

## Additional information

**How to cite this article:** Zhao, W. *et al*. Multi-localization transport behaviour in bulk thermoelectric materials. *Nat. Commun.* 6:6197 doi: 10.1038/ncomms7197 (2015).

## Supplementary Material

Supplementary Information Supplementary Figures 1-5

## References

[b1] Bell L. E. Cooling, heating, generating power, and recovering waste heat with thermoelectric systems. Science 321, 1457–1461 (2008). 18787160 10.1126/science.1158899

[b2] Snyder G. J. & Toberer E. S. Complex thermoelectric materials. Nat. Mater. 7, 105–114 (2008). 18219332 10.1038/nmat2090

[b3] Biswas K. . High-performance bulk thermoelectrics with all-scale hierarchical architectures. Nature 489, 414–418 (2012). 22996556 10.1038/nature11439

[b4] Hsu K. F. . Cubic AgPb_*m*_SbTe_2+*m*_: Bulk thermoelectric materials with high figure of merit. Science 303, 818–821 (2004). 14764873 10.1126/science.1092963

[b5] Dresselhaus M. S. . New directions for low-dimensional thermoelectric materials. Adv. Mater. 19, 1043–1053 (2007).

[b6] Poudel B. . High-thermoelectric performance of nanostructured bismuth antimony telluride bulk alloys. Science 320, 634–638 (2008). 18356488 10.1126/science.1156446

[b7] Morelli D. T., Jovovic V. & Heremans J. P. Intrinsically minimal thermal conductivity in cubic I-V-VI_2_ semiconductors AgSbTe_2_ and AgBiSe_2_. Phys. Rev. Lett. 101, 035901-1–035901-4 (2008). 18764265 10.1103/PhysRevLett.101.035901

[b8] Sales B. C., Mandrus D. & Williams R. K. Filled skutteruditeantimonides: a new class of thermoelectric materials. Science 272, 1325–1328 (1996). 8662465 10.1126/science.272.5266.1325

[b9] Siemens M. E. . Quasi-ballistic thermal transport from nanoscale interfaces observed using ultrafast coherent soft X-ray beams. Nat. Mater. 9, 26–30 (2010). 19898462 10.1038/nmat2568

[b10] Zhao W. Y. . Enhanced thermoelectric performance via randomly arranged nanopores: excellent transport properties of YbZn_2_Sb_2_ nanoporous materials. Acta Mater. 60, 1741–1746 (2012).

[b11] Liu H. L. . Copper ion liquid-like thermoelectric. Nat. Mater. 11, 422–425 (2012). 22406814 10.1038/nmat3273

[b12] Delaire O. . Giant anharmonic phonon scattering in PbTe. Nat. Mater. 10, 614–619 (2011). 21642983 10.1038/nmat3035

[b13] Pei Y. Z. . Convergence of electronic bands for high performance bulk thermoelectric. Nature 473, 66–69 (2011). 21544143 10.1038/nature09996

[b14] Liu W. . Convergence of conduction bands as a means of enhancing thermoelectric performance of n-type Mg_2_Si_1−x_Sn_x_ solid solutions. Phys. Rev. Lett. 108, 166601–166605 (2012). 22680741 10.1103/PhysRevLett.108.166601

[b15] Rhyee J. S. . Peierls distortion as a route to high thermoelectric performance in In_4_Se_3_-delta crystals. Nature 459, 965–968 (2009). 19536260 10.1038/nature08088

[b16] Heremans J. P. . Enhancement of thermoelectric efficiency in PbTe by distortion of the electronic density of states. Science 321, 554–557 (2008). 18653890 10.1126/science.1159725

[b17] Ahmad S., Hoang K. & Mahanti S. D. *Ab Initio* study of deep defect states in narrow band-gap semiconductors: Group III impurities in PbTe. Phys. Rev. Lett. 96, 056403-1–056403-4 (2006). 16486963 10.1103/PhysRevLett.96.056403

[b18] Lee J.-H., Wu J. & Grossman J. C. Enhancing the thermoelectric power factor with highly mismatched isoelectronic doping. Phys. Rev. Lett. 104, 016602-1–066602-4 (2010). 20366377 10.1103/PhysRevLett.104.016602

[b19] Zhou J., Yang R. G., Chen G. & Dresselhaus M. S. Optimal bandwidth for high efficiency thermoelectric. Phys. Rev. Lett. 107, 226601-1–226601-5 (2011). 22182036 10.1103/PhysRevLett.107.226601

[b20] Nolas G. S., Cohn J. L. & Slack G. A. Effect of partial void filling on the lattice thermal conductivity of skutterudites. Phys. Rev. B 58, 164–170 (1998).

[b21] Uher C. inRecent Trends in Thermoelectric Materials Research I, Semiconductors and Semimetals ed. Tritt T. M. Vol. 69139–253Academic Press (2001).

[b22] Nolas G. S., Kaeser M., Littleton R. T. & Tritt T. M. High figure of merit in partially filled ytterbium skutterudite materials. Appl. Phys. Lett. 77, 1855–1857 (2000).

[b23] Morelli D. T., Meisner G. P., Chen B. X., Hu S. Q. & Uher C. Cerium filling and doping of cobalt triantimonide. Phys. Rev. B 56, 7376–7383 (1997).

[b24] Chen L. D. . Anomalous barium filling fraction and *n*-type thermoelectric performance of Ba_*y*_Co_4_Sb_12_. J. Appl. Phys. 90, 1864–1868 (2001).

[b25] Zhao W. Y. . Synthesis and high temperature transport properties of barium and indium double-filled skutterudites Ba_*x*_In_*y*_Co_4_Sb_12−*z*_. J. Appl. Phys. 102, 113708-1–113708-6 (2007).

[b26] Pei Y. Z. . Improving thermoelectric performance of caged compounds through light-element filling. Appl. Phys. Lett. 95, 042101-1–042101-3 (2009).

[b27] Zhao W. Y. . Enhanced thermoelectric performance in barium and indium double-filled skutterudite bulk materials via orbital hybridization induced by indium filler. J. Am. Chem. Soc. 131, 3713–3720 (2009). 19245204 10.1021/ja8089334

[b28] Shi X. . Multiple-filled skutterudites: high thermoelectric figure of merit through separately optimizing electrical and thermal transports. J. Am. Chem. Soc. 133, 7837–7846 (2011). 21524125 10.1021/ja111199y

[b29] Mei Z. G., Yang J., Pei Y. Z., Zhang W. & Chen L. D. Alkali-metal-filled CoSb_3_ skutterudites as thermoelectric materials: theoretical study. Phys. Rev. B 77, 045202-1–045202-8 (2008).

[b30] Sales B. C., Chakoumakos B. C. & Mandrus D. Thermoelectric properties of thallium-filled skutterudites. Phys. Rev. B 61, 2475–2481 (2000).

[b31] Hermann R. P. . Einstein oscillators in thallium filled antimony skutterudites. Phys. Rev. Lett. 90, 135505-1–135505-4 (2003). 12689305 10.1103/PhysRevLett.90.135505

[b32] Qiu Y. T. . Charge-compensated compound defects in Ga-containing thermoelectric skutterudites. Adv. Funct. Mater. 23, 3194–3203 (2013).

[b33] Tang Y. L. . Phase diagram of In–Co–Sb system and thermoelectric properties of In-containing skutterudites. Energy Environ. Sci. 7, 812–819 (2014).

[b34] He T., Chen J. Z., Rosenfeld H. D. & Subramanian M. A. Thermoelectric properties of indium-filled skutterudites. Chem. Mater. 18, 759–762 (2006).

[b35] Harnwunggmoung A. . Enhancement of thermoelectric properties of CoSb_3_-based skutterudites by double filling of Tl and In. J. Appl. Phys. 112, 043509-1–043509-6 (2012).

[b36] Wang L., Cai K. F., Wang Y. Y., Li H. & Wang H. F. Thermoelectric properties of indium-filled skutterudites prepared by combining solvothermal synthesis and melting. Appl. Phys. A 97, 841–845 (2009).

[b37] Wei P. . Excellent performance stability of Ba and In double-filled skutterudite thermoelectric materials. Acta Mater. 59, 3244–3254 (2011).

[b38] Yu J. . Effect of In impurity on thermoelectric properties of Ba and In double-filled n-type skutterudite materials. J. Electron. Mater. 41, 1395–1400 (2012).

[b39] Peng J. Y. . High temperature thermoelectric properties of double-filled In_*x*_Yb_*y*_Co_4_Sb_12_ skutterudites. J. Appl. Phys. 105, 084907-1–084907-5 (2009).

[b40] Grytsiv A., Rogl P., Michor H., Bauer E. & Giester G. In_y_Co_4_Sb_12_ skutterudite: Phase equilibria and crystal structure. J. Electron. Mater. 42, 2940–2952 (2013).

[b41] Eilertsen J., Rouvimov S. & Subramanian M. A. Rattler-seeded InSb nanoinclusions from metastable indium-filled In_0.1_Co_4_Sb_12_ skutterudites for high-performance thermoelectric. Acta Mater. 60, 2178–2785 (2012).

[b42] Li H., Tang X. F., Zhang Q. J. & Uher C. High performance In_*x*_Ce_*y*_Co_4_Sb_12_ thermoelectric materials with *in situ* forming nanostructured InSb phase. Appl. Phys. Lett. 94, 102114-1–102114-3 (2009).

[b43] Chen D. L., Li C. S., Zhu Z. G., Fan J. W. & Wei S. Q. Interface effect of InSb quantum dots embedded in SiO_2_ matrix. Phys. Rev. B 72, 075341-1–075341-7 (2005).

[b44] Zhao X. Y. . Synthesis and thermoelectric properties of Sr-filled skutterudite Sr_*y*_Co_4_Sb_12_. J. Appl. Phys. 99, 053711-1–053711-4 (2006).

[b45] Li H., Tang X. F., Su X. L., Zhang Q. J. & Uher C. Nanostructured bulk Yb_*x*_Co_4_Sb_12_ with high thermoelectric performance prepared by the rapid solidification method. J. Phys. D Appl. Phys. 42, 145409-1–145409-9 (2009).

[b46] Bai S. Q. . Enhanced thermoelectric performance of dual-element-filled skutterudites Ba_*x*_Ce_*y*_Co_4_Sb_12_. Acta Mater. 57, 3135–3139 (2009).

[b47] Shi X. . Low thermal conductivity and high thermoelectric figure of merit in n-type Ba_*x*_Yb_*y*_Co_4_Sb_12_ double-filled skutterudites. Appl. Phys. Lett. 92, 182101-1–182101-3 (2008).

[b48] Lamberton G. A. Jr., Tedstrom R. H., Tritt T. M. & Nolas G. S. Thermoelectric properties of Yb-filled Ge-compensated CoSb_3_ skutterudite materials. J. Appl. Phys. 97, 113715-1–113715-5 (2005).

[b49] Kuznetsov V. L., Kuznetsova L. A. & Rowe D. M. Effect of partial void filling on the transport properties of Nd_*x*_Co_4_Sb_12_ skutterudites. J. Phys Condens. Matter. 15, 5035–5048 (2003).

[b50] Rehr J. J. & Albers R. C. Theoretical approaches to X-ray absorption fine structure. Rev. Mod. Phys. 72, 621–654 (2000).

[b51] Payne M. C., Teter M. P., Allan D. C., Arias T. A. & Joannopoulos J. D. Iterative minimization techniques for *ab initio* total-energy calculations: molecular dynamics and conjugate gradients. Rev. Mod. Phys. 64, 1045–1097 (1992).

