# Supplementary material for: Multi-localization transport behaviour in bulk thermoelectric materials
Source: Nat Commun. 2015 Feb 4;6:6197. doi: 10.1038/ncomms7197 (PMC4327545; doi:10.1038/ncomms7197)
Supplement: Supplementary Information — Supplementary Figures 1-5 [file ncomms7197-s1.pdf]

SUPPLEMENTARY FIGURES

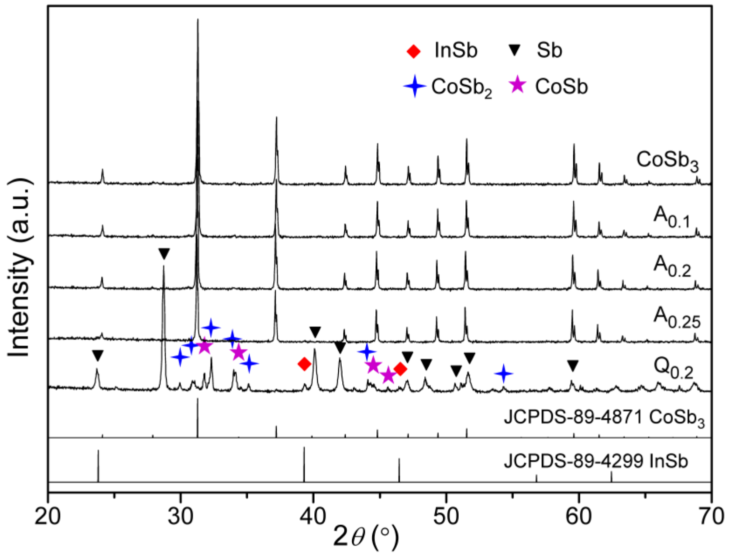

Supplementary Figure 1 | XRD of $CoSb_3$ and In-doped samples. The quenched $In_{0.2}Co_4Sb_{12}$ is symbolized with "$Q_{0.2}$". The annealed $In_xCo_4Sb_{12}$ (x=0.1,0.2, and 0.25) is symbolized with "A_x". It can be seen that all the A_x samples are composed of single-phase skutterudite, however, the quenched $Q_{0.2}$ sample consists of Sb, CoSb, $CoSb_2$, and InSb.

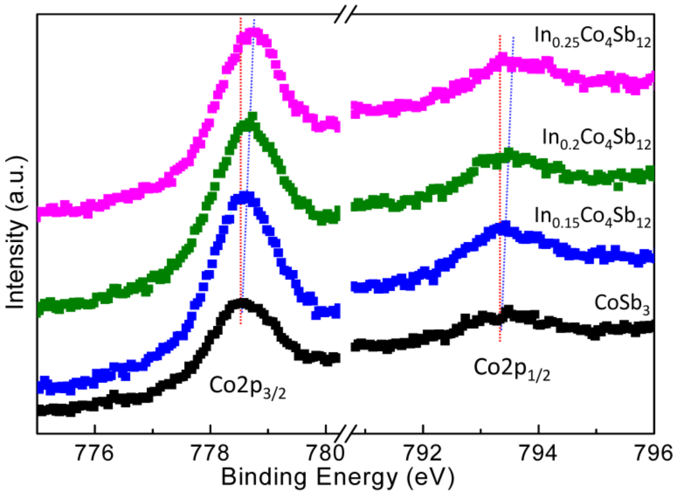

Supplementary Figure 2 | XPS spectra of Co 2$p_{3/2}$ and 2$p_{1/2}$ of $CoSb_3$ and $In_xCo_4Sb_{12}$. The chemical shift as shown in Fig. 4 is obtained under CAE mode with pass energy of 25eV, step size of 0.05eV and 128 scans with a Thermo VG Multilab 2000 spectrometer at Key Laboratory of Catalysis and Materials Science of the State Ethnic Affair Commission & Ministry of Education of South-Central University for Nationalities. However, the maximum chemical shift of about 0.2 eV is less than the energy resolution of XPS (about 0.47 eV). To clarify whether the chemical shift as shown in Fig. 4 can be repeated, the XPS spectra of Co 2$p_{3/2}$ and 2$p_{1/2}$ core levels of $CoSb_3$ and $In_xCo_4Sb_{12}$ have been again recorded under CAE mode with energy of 30eV, step size of 0.05eV, and 15 scans with a Thermo Fisher ESCALAB 250Xi instrument at Wuhan University and the results are shown in Supplementary Figure 2. It can be seen that the maximum chemical shift reaches 0.25eV for $In_{0.25}Co_4Sb_{12}$. To examine whether the chemical shift can be easily repeated, the scans were intentionally decreased from 128 scans to 15 scans during the following experiment at Wuhan University. As compared with Fig. 4, the unsmooth spectra in Supplementary Figure 2 are attributed to the great decrease in the scans. Although the maximum chemical shift from Supplementary Figure 2 (0.25eV) is still less than the energy resolution of XPS, it can be drawn a conclusion according to the experimental results that the chemical shift of XPS spectra of Co 2$p_{3/2}$ and 2$p_{1/2}$ core levels of In-filled $CoSb_3$ is a repeated phenomenon.

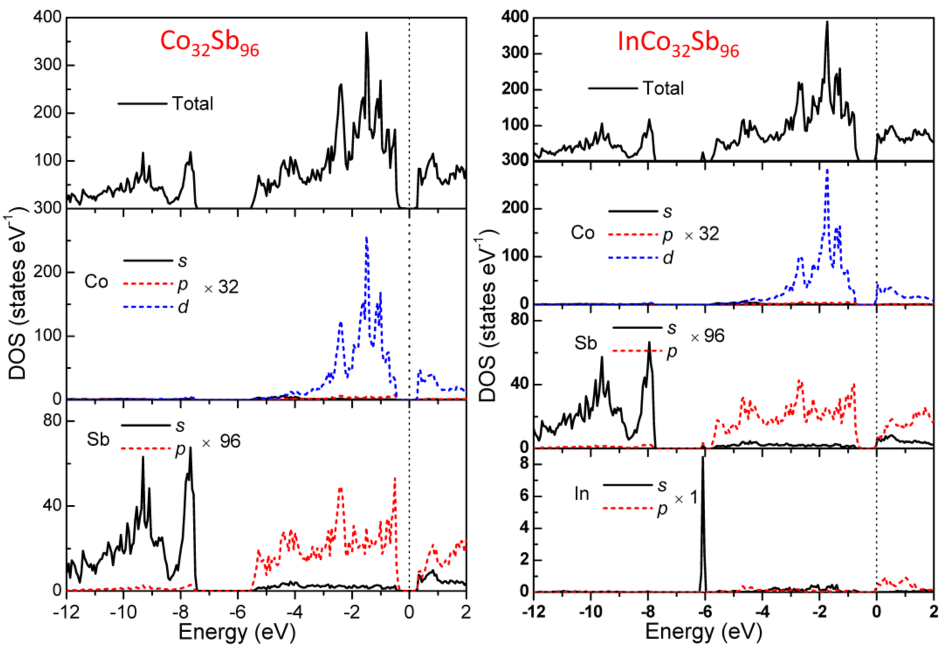

Supplementary Figure 3 | Total DOS of $CoSb_3$ and $In_{0.125}Co_4Sb_{12}$ and partial DOS for Co, Sb and In atoms. It can be seen that the total DOS of $CoSb_3$ and In-filled $In_{0.125}Co_4Sb_{12}$ near valence band maximum (VBM) and conduction band minimum (CBM) mainly stem from Co 3*d* electrons and Sb 5*p* electrons. An extra peakof the partial DOS of Co 3*d* and Sb 5*p* electrons near 0.31eV for $In_{0.125}Co_4Sb_{12}$ exactly corresponds with the highest peak of the partial DOS of In 5*p* electrons. The partial DOS ofIn 5*s* electrons are distributed about 1.0 eV below the Fermi level, indicating that all 5*s* electrons are confined at the deep locations of valence band and have no contribution to the *n*. Although there are a few 5*p* orbitals of In filler below Fermi level, the partial DOS of 5*p* electrons of In filler are mainly distributed above and near the Fermi level, suggesting that 5*p* electron is almost lost in In-filled $CoSb_3$. The electronic states of In impurity in $CoSb_3$ clearly show that the effective charge of In filler is smaller than but very close to +1.

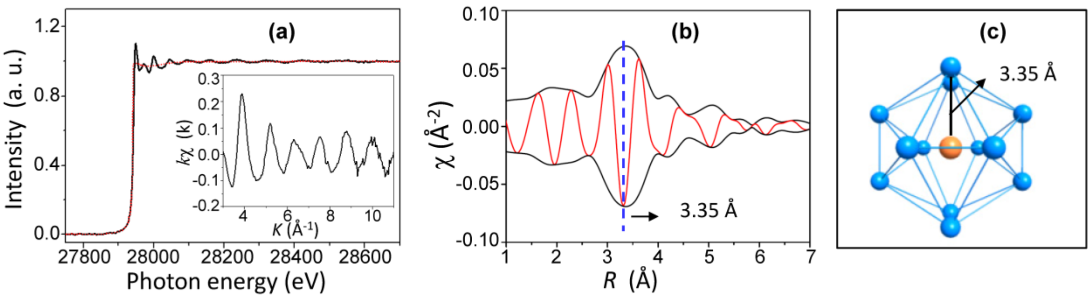

Supplementary Figure 4 | The In-Sb weak covalent bond in In-filled $CoSb_3$. (a) The In *K*-edge Extended X-ray absorption fine structure (EXAFS) experimental spectrum and *k*-weighted EXAFS spectrum for $In_{0.18}Co_4Sb_{12}$, **(b)** Radial distribution function obtained through Fourier transformation of *k*-weighted EXAFS spectrum, and **(c)** In-filled icosahedronof In-filled $CoSb_3$. It shows that the length of In-Sb bond is about 3.35 Å in $In_{0.18}Co_4Sb_{12}$ while it is only about 2.81 Å in InSb reported in the reference [43], which is very close to 3.36 Å in $In_{0.2}Co_4Sb_{12}$ reported in the reference [34].

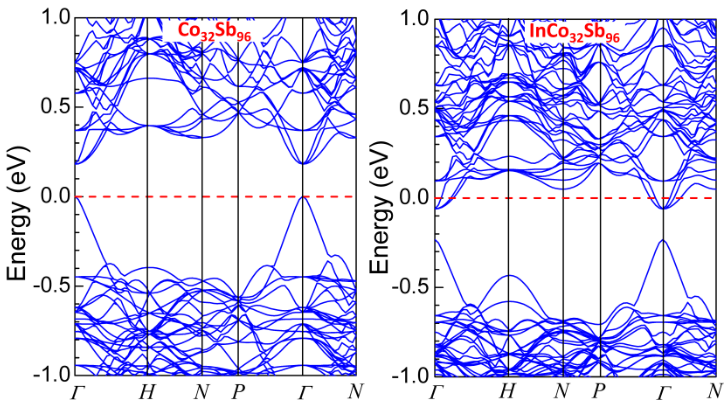

Supplementary Figure 5 | Theband structure of $CoSb_3$ and $In_{0.125}Co_4Sb_{12}$. The 2×2×2 supercells were calculated with projector augmented wave method implemented in CASTEP package based on density functional theory (DFT). Compared to $CoSb_3$ as shown in the right side, the Fermi level of $In_{0.125}Co_4Sb_{12}$ as shown in the left side is migrated into conduction bands, the energy gap between Fermi level and CBM at *H*, *N*, and *P* points with high symmetry is significantly decreased from 0.45~0.35 eV for $CoSb_3$ to 0.12~0.03 eV for $In_{0.125}Co_4Sb_{12}$. As a result, the DOS of VBM is significantly decreased while the DOS of CBM is remarkably increased. Namely, there is an asymmetric distribution of DOS near the Fermi level of In-filled $CoSb_3$ beneficial to obtaining a large α.
